# Depression and anxiety symptoms and perceived stress in health professionals in the context of COVID‐19: Do adverse childhood experiences have a modulating effect?

**DOI:** 10.1002/brb3.2452

**Published:** 2021-12-15

**Authors:** Alberto Fernández‐Arana, Adriel Olórtegui‐Yzú, Johann M. Vega‐Dienstmaier, Manuel J. Cuesta

**Affiliations:** ^1^ Instituto de Neurociencias Aplicadas Lima Peru; ^2^ Universidad Nacional Mayor de San Marcos Facultad de Medina de San Fernando Lima Peru; ^3^ Instituto Nacional Cardiovascular ‐ INCOR ‐ EsSalud Lima Peru; ^4^ Universidad Peruana Cayetano Heredia Facultad de Medicina Alberto Hurtado Lima Peru; ^5^ Department of Psychiatry Complejo Hospitalario de Navarra Pamplona Spain; ^6^ Navarra Institute for Health Research (IdiSNA) Mental health area Pamplona Spain

**Keywords:** adverse childhood experiences, anxiety, COVID‐19, depression, health professionals, SARS‐CoV‐2

## Abstract

**Background:**

Adverse childhood experiences (ACE) have a great impact on mental health outcomes of adults. However, little is known whether ACE may act as modulators of the mental health of health professionals caring for patients with COVID‐19.

**Methods:**

Data were collected through an online cross‐sectional survey administered to health professionals in Lima (Peru) between May and July 2020. The survey included standardized self‐assessment instruments for anxiety, depression, acute stress (AS) and history of ACE.

**Results:**

A total of 542 health professionals completed the survey. Caring for patients with COVID‐19 was significantly associated with depression and anxiety and when caring for patients with COVID‐19 was combined with a history of early sexual abuse, its effect on the risk of anxiety increased (OR = 7.71, *p* = .010). Mental health problems were associated with female gender in almost all the analyses and with the majority of ACEs.

**Conclusions:**

Health workers in the context of the COVID‐19 pandemic presented a high risk of mental health disorders. Antecedents of sexual abuse acted as a potentiating factor of anxiety in professionals providing COVID‐19 care. These findings suggest that the burden of ACE modulates mental health problems in health professionals during the pandemic.

## Graphical abstract

‐ In the context of the pandemic, health workers have a higher risk of mental disorders.

‐ Early sexual abuse increases the risk of anxiety in frontline workers.

‐ The experience of ACE modulates the presence of mental disorders in health workers.

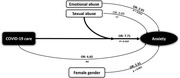

## 1 INTRODUCTION

As of January 10, the COVID‐19 pandemic has led to 1,932,948 deaths worldwide and 30,049 in Peru (Johns Hopkins University of Medicine, n.d.). The rates of professionals with COVID‐19 vary among countries, but it is worth noting that the number is substantial. In Latin America, the deaths of 2741 physicians have been reported dead, including 257 in Peru (Colegio Médico del Peru, n.d.).

In all countries, health systems, including hospitals and health centers, had to be reorganized to reflect new care priorities for patients with COVID‐19. These adaptations within health systems forced changes and disrupted the usual work dynamics of health professionals; in many cases, these adaptations caused work changes, such as the transfer of professionals from their usual practice to first‐line care, and, in many cases, forced a reorganization of all care flow patterns (Moreno et al., 2020). In addition, the lack of adequate resources and protective gear to guard against possible infections generated high rates of COVID‐19 infections among professionals (Erquicia et al., 2020). Changes in routine practice related to COVID‐19, the high risk of COVID‐19 infection, and uncertainties in decision‐making are new workplace stressors that can potentially affect the mental health of professionals and are risk factors for the development of psychological distress, anger, and depression (Sanghera et al., 2020; Serrano‐Ripoll et al., 2020).

There is strong evidence suggesting that trauma experienced during childhood or adolescence can have a particular etiological significance in determining the mental health outcomes of adults (McKay et al., 2020). However, little is known about the effects of early traumatic experiences during childhood or adolescence that can modulate health professionals’ responses to new stressors that have arisen since the beginning of the COVID‐19 pandemic.

There is consistent evidence in the literature that an individual's resistance to persistent stress is closely related to his or her personal history of adverse childhood experiences (ACEs) and is associated with lower resilience (Ayyala et al., 2020; Barnes et al., 2020; Humphreys & Zeanah, 2015; Kessler et al., 2010). Some authors find that ACEs are also associated with an increased risk of anxiety in adulthood (Bandelow et al., 2004; Gal et al., 2011) and with other psychiatric problems, including substance use disorders (Bandelow et al., 2004; Gal et al., 2011; Norman et al., 2012; Pesonen et al., 2007; Räikkönen et al., 2011), personality disorders (Johnson et al., 1999; Lahti et al., 2012), psychosis (Varese et al., 2012), and suicidal tendencies (Dube et al., 2001; Norman et al., 2012). Considering that ACEs can be a predisposing factor for anxiety disorders, depression, and pathological stress (Norman et al., 2012) and that the COVID‐19 pandemic is a strong and constant stressor (Shigemura et al., 2020), the probability that health workers who have experienced ACEs will suffer from these pathologies is very high (Siu, 2008; Xiang et al., 2020).

The objective of the study was to evaluate, in health professionals in Lima (Peru), the relationship between mental health problems (anxiety, depression, and perceived stress), and the care of patients with COVID‐19 and early adverse events. Likewise, to evaluate whether early adverse events modulate the relationship the care of patients with COVID‐19 and mental healt problems in these professional.

## 2 METHOD

### 2.1 Sample

Data from a sample of 542 health professionals working in the Lima region, mainly women (64.3%) aged between 24 and 87 years (Table 1), were collected through an online questionnaire using Google Forms. Participants were recruited through advertisements and social media. Due to the lack of health professionals for the care of patients with COVID‐19, all health professionals, regardless of their training, were assigned to work directly or indirectly in the care of these patients. For this reason, the following were included in the study: doctors, nurses, midwives, medical technologists, nutritionists, pharmaceutical chemists, nursing technicians, psychologists, and biologists. The survey was conducted in May and June 2020 at the peak of contagion and mortality in Lima, Peru.

**TABLE 1:** General characteristics of health workers. 2020 Mental Health Survey Lima

Variable	N°	%
Female gender	344	63.5
Marital status		
Married	323	59.6
Single	219	40.4
Professional group		
Physician	302	55.7
Nurse	90	16.6
Midwife	64	11.8
Other	86	15.9
COVID‐19 care	216	39.9
Change in service	138	25.5
Diagnosed with COVID‐19	35	6.5

### 2.2 Measurements

The instrument consisted of 48 questions distributed among seven sections. The questionnaire was anonymous to ensure the privacy and confidentiality of the participants. The structure of the survey did not allow advancing to the next question until the previous question was answered. The variables included personal, occupational, occupational exposure to COVID‐19 and recent COVID‐19 diagnosis, ACE, anxiety, depression, and acute stress (AS).

Exposure to COVID‐19 was measured by two questions that were incorporated into the questionnaire: one asked whether the health professional was involved in the direct care of patients with COVID‐19, and the other asked whether the health worker was moved to a COVID‐19 patient care area different from his/her usual area of work.

For the present study, the ACE data was obtained according to the procedure defined by J. Douglas Bremner in his Early Trauma Inventory Self Report‐Short Form (ETI), which addresses the following types of traumas occurring before the age of 18 years (Posada et al., 2019):

- **General trauma** investigates the occurrence of some type of negative, violent, or traumatic event in which the subject only witnessed violent events.
- **Physical abuse** refers to the experience of physical abuse in childhood.
- **Emotional abuse** refers to the experience of emotional abuse in childhood.
- **Sexual abuse** refers a history of abuse, mistreatment, trauma, or physical invasion of a sexual nature.

Due to limitations of the survey, we only included four questions addressing each type of trauma (See Appendix). The ETI has been validated in Colombia (Posada et al., 2019).

Anxiety in the last 2 weeks was measured with the generalized anxiety disorder scale, version 7 (GAD‐7), which has been validated in Peru (Zhong et al., 2015); the presence of anxiety is determined when the score is > 4 (Spitzer et al., 2006). Depression in the last 2 weeks was assessed with the Patient Health Questionnaire version 9 (PHQ‐9) (“Cuestionario sobre la salud del Paciente (PHQ‐9),” n.d.), which was also validated in Peru (Villarreal‐Zegarra et al., 2019); the presence of depression is determined when the scores is > 4 (Kisely et al., 2020). Acute stress in the last month was measured with the Global Perceived Stress Scale version 10 (PSS‐10) (Cohen et al., 1983). The median value of the obtained distribution was used as a cutoff point; values of 16 or higher were considered indicative of the presence of AS, and values less than 16 were considered indicative of the absence of AS (Guzmán‐Yacaman & Reyes‐Bossio, 2018). The PSS has been validated in our setting (Raygada Zolezzi, 2019).

### 2.3 Statistical analysis

The power and size of the sample was estimated before the study using Epidat v 3.1 (Junta de Gobierno de Galicia, n.d.) and considering maximum variability, a significance of 0.05, an accuracy of 5%, and a data loss of 10%. The minimum sample size required was 430 workers.

The statistical analysis of the data was performed with the statistical software Stata v 16. First, the frequency distributions and summary measures were analyzed, allowing us to identify the patterns and distribution of the variables. Second, we examined the relationships between the dependent variables (depression, anxiety, and acute stress) and the independent variables (caring for patients with COVID‐19, change in service, being diagnosed with COVID‐19, general trauma, physical abuse, psychological trauma, sexual abuse, gender, and age) using bivariate and multivariate logistic regression analyses. In the logistic regression analysis, two models were used: 1) a model without interactions; 2) a model with interactions between caring for patients with COVID‐19 and a) each type of ACE, b) gender, and c) age. The latter model was used to evaluate whether the effect of caring for patients with COVID‐19 on mental health was modified by ACEs, gender and/or age.

In case of finding a significant interaction between an ACE and the care of patients with COVID, stratified analyzes were performed, separating the sample into individuals with and without the ACE; and on the other hand, dividing it into health personnel who attend and who do not attend to patients with COVID‐19.

Cronbach's α test was performed to determine the internal consistency of the questions on the questionnaire concerning depression, anxiety, and stress.

### 2.4 Ethics

The study procedures were conducted in accordance with the ethical standards for human experimentation. The study was approved by the “Carlos Alberto Peschiera Carrillo” National Cardiovascular Ethics Committee ‐ INCOR ‐ EsSalud (Certificate of Approval 09/2020).

## 3 RESULTS

A total of 542 health professionals completed the online questionnaire. The dropout rate related to abandoned or incomplete questionnaires was not estimated since the web platform did not allow it. The demographic characteristics of the health workers are summarized in Table 1.

The Cronbach's α values of the PHQ‐9, GAD‐7, and PSS in our sample showed high internal consistency and were 0.9081, 0.9284, and 0.8309, respectively.

The mean age of the respondents was 49.2 years (minimum 24 and maximum 87). A total of 63.5% were women. The women had a mean age of 47.5 years, and the men had a mean age of 52.3 years; the difference was statistically significant (*F* = 17.269, *p* < .001, df = 541). The number of participating physicians (55.7%) was larger than the number of nurses (16.6%) and midwives (11.8%).

The prevalence of anxiety, depression, and acute stress in the total sample was 54.2%, 44.5%, and 50.7%, respectively. In all three domains, women had significantly higher scores than men (Table 2).

**TABLE 2:** Distribution of adverse childhood experiences, anxiety and depression according to gender. 2020 Mental Health Survey Lima

	Total sample		Men		Women		Statistical difference between men and women	
Variables	N	%	N	%	N	%	0R	*p*
Anxiety	294	54.2	77	38.9	217	63.1	2.69 (1.87–3.85)	<.001
Mild‐severe depression	241	44.5	64	32.3	177	51.5	2.22 (1.54–3.20)	<.001
Acute stress	242	50.7	78	39.4	197	57.3	2.06 (1.44–2.94)	<.001
General trauma	383	70.7	135	68.2	248	72.1	1.21 (0.82–1.76)	NS
Emotional abuse	324	59.8	99	50.0	225	65.4	1.89 (1.32–2.70)	<.001
Physical abuse	170	31.4	65	32.8	105	30.5	0.90 (0.62–1.31)	NS
Sexual abuse	56	10.3	9	4.5	47	13.7	3.23 (1.60–6.94)	<.001

Female health professionals had a significantly higher prevalence of emotional abuse than males did (OR = 1.89, *p* < .001). Men reported a significantly higher prevalence of sexual abuse than women (OR = 3.23, *p* < .001) (Table 2). There were no significant differences between genders in general trauma and physical abuse.

In the context of COVID‐19, 25.5% of health workers were assigned to another service for pandemic‐related reasons, while 39.9% of health workers treated patients with COVID‐19. Of these workers, 6.5% reported having been diagnosed with COVID‐19.

### 3.1 Bivariate and multivariate analysis

Anxiety was significantly more frequent in younger and female professionals (Tables 2 and 3). Anxiety was also strongly associated with reports of experiences of general trauma, emotional abuse and sexual abuse (but not physical abuse), as well as younger age, female gender and COVID care. However, the effect size was small for all variables (Table 3).

**TABLE 3:** Association between anxiety and the different variables according to the bivariate analyses and multivariate models with and without interactions

Anxiety	Bivariate analysis		Model adjusted to include all variables		Model with interaction			
Variable	OR	*p*	All variables	*p*	Direct effect	*p*	With COVID care	*p*
General trauma	1.55 (1.07–2.25)	.021	1.21 (0.78–1.86)	NS	1.35 (0.79–2.32)	NS	0.63 (0.25–1.58)	NS
Physical abuse	1.36 (0.94–1.96)	NS	0.83 (0.52–1.31)	NS	0.88 (0.50–1.60)	NS	0.80 (0.31–2.09)	NS
Emotional abuse	2.27 (1.60–3.22)	<.001	1.80 (1.17–2.77)	.009	2.01 (1.17–3.44)	.011	0.86 (0.34–2.15)	NS
Sexual abuse	2.79 (1.48–5.23)	<.001	1.95 (0.99–3.82)	NS	0.89 (0.38–2.15)	NS	7.71 (1.62–36.80)	.01
Female gender	2.69 (1.87–3.85)	<.001	2.20 (1.51–3.22)	.004	2.61 (1.60–4.24)	<.001	0.61 (0.27–1.34)	NS
Age*	0.97 (0.96–0.99)	<.001	0.98 (0.97–0.99)	<.001	0.99 (0.97–1.01)	NS	0.99 (0.97–1.01)	NS
Change in service	1.05 (0.74–1.47)	NS	1.00 (0.69–1.44)	NS	1.00 (0.70–1.50)	NS		
COVID care	1.59 (1.12–2.26)	.009	1.18 (0.78–1.78)	NS	4.65 (0.67–32.20)	NS		
COVID diagnosis	1.29 (0.64–2.59)	NS	2.11 (0.82–5.43)	NS	0.96 (0.45–2.10)	NS		

* The model incorporates the age variable using a continuous quantitative scale.

In the logistic regression models adjusted to include all variables, anxiety was significantly associated with female gender (OR = 2.20, *p* = 0.004) and emotional abuse (OR = 2.01 *p* = .011), and age maintained an inverse association (OR = 0.98, *p* < .001).

**FIGURE 1:**
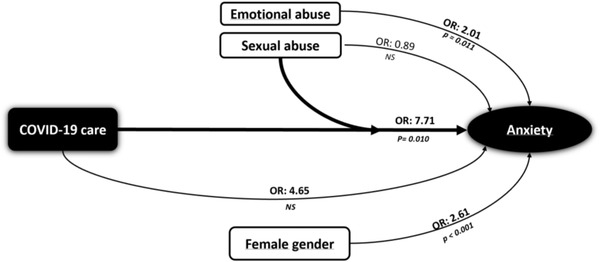
interaction associations between COVID‐19 care and ACE, anxiety, and female gender. Note: The graph shows how the coexistence of childhood sexual abuse and COVID‐19 care was significantly associated with anxiety (OR: 7.71)

In the logistic regression models that introduced each of the traumatic events, including the interaction between COVID‐19 care and the reported intensity of anxiety, it was found that a history of sexual abuse significantly increased self‐reported anxiety in professionals working in COVID‐19 care (OR = 7.71, *p* = .010) (Table 3 and Figure 1).

In the stratified analysis, considering only individuals without a history of sexual abuse (*n* = 486), there was no significant relationship between the care of patients with COVID and anxiety, both in the bivariate analysis (OR = 1.33, 95% CI: 0.92−1.92) as in the one adjusted for sex and age (OR = 1.07, 95% CI: 0.70–1.63); on the other hand, considering only the participants with a history of sexual abuse (*n* = 56), a significant relationship was observed: crude OR = 7.33 (95% CI: 1.76–30.61) and OR adjusted for sex and age = 5.23 (95% CI: 1.17–23.47). Likewise, dividing the sample between the professionals who attended to patients with COVID (*n* = 216) and those who did not (*n* = 326), the first group did show a relationship between a history of sexual abuse and anxiety: crude OR = 7.27 (95 CI %: 2.13–24.76), OR adjusted for age and sex = 6.59 (CI: 1.90–22.85); while there was no significant relationship between those who did not attend to patients with COVID: crude OR = 1.31 (95% CI: 0.58–2.99), OR adjusted for age and sex = 1.04 (95% CI: 0.45–2.43).

### 3.2 Depression

Self‐reported depression was significantly associated with the four types of traumatic experiences, younger age, female gender, and COVID‐19 care (ORs between 0.97 and 3.23, significance *p* = .005 and < .001). The effect size was small for all variables (Table 4).

**TABLE 4:** Association between depression and different variables according to the bivariate analysis and multivariate models with and without interactions

Depression	Bivariate analysis		Model adjusted to include all variables		Model with interaction			
Associated variables	OR	*p*	All variables	*p*	Direct Effect	*p*	With COVID care	*p*
General trauma	1.72 (1.17–2.52)	.005	1.38 (0.89–2.14)	NS	1.44 (0.82–2.52)	NS	0.84 (0.33–2.11)	NS
Physical abuse	1.54 (1.07–2.21)	.002	0.95 (0.61–1.50)	NS	1.10 (0.59–1.90)	NS	0.75 (0.30–1.90)	NS
Emotional abuse	2.07 (1.45–2.95)	<.001	1.38 (0.89–2.13)	NS	1.39 (0.80–2.40)	NS	1.07 (0.42–2.64)	NS
Sexual abuse	3.23 (1.77–5.86)	<.001	2.36 (1.24–4.49)	.008	2.01 (0.83–4.85)	NS	1.51 (0.41–5.59)	NS
Female gender	2.22 (1.54–3.20)	<.001	1.81 (1.23–2.68)	.003	1.96 (1.20–3.24)	.009	0.81 (0.36–1.82)	NS
Age*	0.97 (0.95–0.98)	<.001	0.98 (0.96–0.99)	.005	0.98 (0.96–1.00)	NS	0.98 (0.96–1.02)	NS
Change in service	1.26 (0.89–1.77)	NS	1.22 (0.85–1.77)	NS	1.21 (0.83–1.77)	NS	–.–	
COVID care	1.99 (1.40–2.82)	<.001	1.36 (0.91–2.04)	NS	3.12 (0.46–20.99)	NS	–.–	
COVID diagnosis	1.35 (0.68–2.68)	NS	0.96 (0.46–2.02)	NS	0.98 (0.17–2.28)	NS	–.–	

* The model incorporates the age variable on a continuous quantitative scale.

In the logistic regression models adjusted to include all variables, depression was significantly associated with female gender (OR = 2.20, *p* = .004) and emotional abuse (OR = 2.01, *p* ≤ .011), although the effect size was small. Age also presented an inverse relationship, with younger age associated with an increased risk of depression (OR = 0.98, *p* = .005)

The multivariate analysis with interactions showed that female gender was significantly associated with depression, but there were no significant interactions associating traumatic events with COVID‐19 care and depression in health professionals. (see Table 4)

### 3.3 Acute stress

A self‐reported history of emotional abuse (OR = 2.21, *p* = .008) or sexual abuse OR = 3.23, *p* < .001) was statistically significantly associated with AS. Professional women presented higher scores for AS (OR = 2.69, *p* < .001) as did younger professionals overall (both men and women) (OR = 0.97, *p* = .020). No significant associations were found between the variable COVID‐19 care and the intensity of AS.

In both the logistic regression models that included all variables and the logistic regression model with interactions, no significant interactions of ACEs were found for the association between COVID‐19 care and current stress. Age maintained an inverse relationship that was statistically significant, and female gender was associated with the presence of AS (OR = 1.73, *p* = .004) (see Table 5).

**TABLE 5:** Association between acute stress and different variables according to the bivariate analysis and multivariate models with and without interactions

Acute stress	Bivariate analysis		Model adjusted to include all variables		Model with interaction			
Associated variables	OR	*p*	All variables	*p*	Direct Effect	*p*	With COVID care	*p*
General trauma	1.14 (0.79–1.65)	NS	0.89 (0.58–1.37)	NS	1.03 (0.61–1.74)	NS	0.66 (0.27–1.64)	NS
Physical abuse	1.31 (0.91–1.88)	NS	0.91 (0.58–1.42)	NS	0.97 (0.54–1.71)	NS	0.86 (0.34–2.13)	NS
Emotional abuse	1.89 (1.34 −2.68)	<.001	1.60 (1.05–2.46)	NS	1.56 (0.92–2.65)	NS	1.09 (0.44–2.67)	NS
Sexual abuse	2.21 (1.23–3.99)	.008	1.68 (0.90–3.15)	NS	1.41 (0.58–3.45)	NS	1.39 (0.39–4.96)	NS
Female gender	2.06 (1.44–2.94)	<.001	1.73 (1.19–2.52)	.004	1.58 (0.98–2.54)	NS	1.24 (0.58–2.71)	NS
Age*	0.98 (0.96–0.99)	<.001	0.98 (0.96–0.99)	.014	0.98 (0.96–1.00)	.020	1.01 (0.98–1.05)	NS
Change in service	1.26 (0.89–1.78)	NS	1.28 (0.89–2.52)	NS	1.31 (0.91–1.89)	NS	–.–	
COVID care	1.18 (0.84–1.67)	NS	0.83 (0.56–1.24)	NS	0.57 (0.01–3.70)	NS	–.–	
COVID diagnosis	1.70 (0.84–3.45)	NS	1.44 (0.69–3.01)	NS	1.39 (0.66–2.94)	NS	–.–	

* The model incorporates the age variable using a continuous quantitative scale.

## 4 DISCUSSION

The main results according to the objectives of the study were as follows. First, our study revealed a high prevalence of self‐reported mental health symptoms among health workers who treated patients with COVID‐19 during the pandemic's period of greatest contagion and mortality in Lima, Peru. Overall, 52.2%, 44.5%, and 50.7% of all participants reported symptoms of depression, anxiety, and acute stress, respectively, and being female significantly increased the rates of mental symptoms. Second, self‐reported exposure to traumatic experiences during childhood or adolescence, particularly sexual abuse, moderated the association between care of COVID‐19 patients and anxiety symptoms.

To our knowledge, this is the first study to examine the role of ACEs as a possible mediating factor in the experience of mental health problems during the COVID‐19 pandemic.

Almost half of our workers indicated experiencing depression, anxiety or stress in the self‐reported questionnaires, which coincides with a recent study of health workers exposed to COVID‐19 in Spain (Alonso et al., 2020). In addition, these results coincide with a meta‐analysis of studies on physical and/or mental health in health workers infected by and/or exposed to severe acute respiratory syndrome (SARS), Middle East respiratory syndrome (MERS) and COVID‐19 (de Pablo et al., 2020). A 6‐week study that explored the mental health of health personnel exposed to COVID‐19 in Canada (Mrklas et al., 2020) again showed high prevalence of anxiety (47.0%), depression (47.0%), and AS (85.6%) (de Pablo et al., 2020). A study in China (Lai et al., 2020) showed that anxiety and depression had prevalence of 44.6% and 50.4%, respectively. Other studies confirm our observations, reporting high prevalence of anxiety (Huang & Zhao, 2020; Huang et al., 2020), depression, (Huang & Zhao, 2020), and AS (Abdessater et al., 2020; Huang et al., 2020). Although these findings can be interpreted as a response to stress resulting from exposure and a high probability of COVID‐19 transmission, we must consider other aspects that could explain them, for example, the implementation of confinement measures, social distancing, the stage of the pandemic in which the study is conducted (at the beginning of the pandemic, in the middle of the pandemic or post pandemic) (Hale et al., 2020), the participants’ degree of knowledge regarding facing this type of health emergency, the lack of supplies and resources, among other factors (Cai et al., 2020). A meta‐analysis of studies conducted in the general population during the COVID‐19 pandemic (Salari et al., 2020) showed that the population also shows also high prevalence of anxiety, depression and AS (31.9%, 33.7%, and 29.6%, respectively), which confirms the need to evaluate other intervening factors.

Regarding the association of COVID‐19 care with a higher prevalence of anxiety and depression symptoms, and AS, our results were consistent with recent studies (Abdessater et al., 2020; Alonso et al., 2020; Cai et al., 2020) that showed an increased risk of mental health disorders in primary care. On the other hand, another study (Zhang et al., 2020) compared the mental health symptoms of medical and nonmedical workers in China after COVID‐19 and found a higher prevalence and greater severity of insomnia, anxiety, depression, and somatization in medical workers. On the other hand, another study (Lu et al., 2020) that compared mental health conditions in front‐line medical workers with those in administrative care workers reported that the former were more likely to perceive fear, anxiety, and depression (1.4 times and 2 times more likely, respectively). Finally, a large online survey conducted after the COVID‐19 pandemic (Huang & Zhao, 2020) failed to find differences in the rates of anxiety and depression between health workers and other workers but found a higher prevalence of sleep disruption in the former group. We must add that variability in conditions such as rapid access to information, training, protocols, personal protective gear, and other occupational and/or social supports can reduce or worsen the explored symptoms (Cai et al., 2020; Kisely et al., 2020; Lim et al., 2018; Ripp et al., 2020; Taylor, 2019).

Regarding ACEs, our data are consistent with the results of a meta‐analysis (LeMoult et al., 2019) in which ACEs were associated with a greater risk of developing depression before the age of 18 years, with an OR of 2.50; this association was strongest for emotional abuse. Another meta‐analysis (Li et al., 2016) confirmed the above finding, reporting a higher risk for anxiety and depression (ORs of 2.7 and 2.0, respectively) with sexual abuse, physical abuse, and neglect being the most associated ACEs (ORs of 2.66, 2.0, and 1.74, respectively).

The results we obtained regarding the gender variable are confirmed by studies that identified that women are more vulnerable to depression, stress and posttraumatic stress disorder than men are (Sareen et al., 2013). Other recent studies confirm that the prevalence of anxiety, depression, and stress during the COVID‐19 pandemic was higher in women than in men (Liu et al., 2020; Moghanibashi‐Mansourieh, 2020; Wang et al., 2021; Zhou et al., 2020).

Finally, our findings regarding how ACEs increase the risk of anxiety and AS, added to the heightened anxiety of workers who are exposed to COVID‐19 while providing front‐line care, coincides with a study conducted in Australia (Kim et al., 2020) in which the perceived risk of COVID‐19 infection was associated with greater depressive symptoms during the first 6 weeks of quarantine among adults with a history of childhood trauma. Another study conducted among Native Americans (John‐Henderson & Ginty, 2020) concluded that childhood trauma predicted greater increases in COVID‐19‐related psychological stress.

Although experiences of abuse during childhood and adolescence can take multiple forms (Higgins & McCabe, 2001), there is strong evidence that child sexual abuse can have specific and persistent consequences for the development of mental disorders in adulthood (Ehring et al., 2014). In particular, it has been shown that childhood sexual abuse in females alters sensitivity to the depressive effects of stressful life events in adulthood (Kendler et al., 2004). Thus, these extreme past experiences can modulate coping strategies in the face of new agents of stress in adult life (Parry & Simpson, 2016).

Growing evidence suggests that mental health disorders are likely the result of a combination of genetic and environmental factors that interact over time. This means that early factors, such as certain genetic polymorphisms, can cause greater brain vulnerability to environmental factors (for example, stress or drugs) at the end of adolescence or young adulthood. In addition, there is solid evidence supporting the fact that childhood trauma leads to an increased risk of subsequent mental illness, fulfilling many of the criteria for the demonstration of causality in the epidemiological association (McKay et al., 2020). Some authors have argued that ACEs induce sensitization to stress in individuals who have higher levels of childhood traumas by inducing a lower tolerance of stress upon exposure to subsequent stressful events (Hammen et al., 2000; Parry & Simpson, 2016). This, in turn, could favor an increase in the stress response that affects the development, structure, and function of the brain and the physiology of other systems, such as the endocrine and immune systems (Clemens et al., 2018). Taken together, ACEs could pave the way for the adaptation of abnormal coping styles by health workers when faced with new stressors for health workers, such as COVID‐19.

It is proposed that these findings be considered to fine‐tune mental health intervention strategies in health workers who are exposed to multiple stressors in their own work and who, having a personal history of adverse childhood experiences, could be more likely to suffer disorders. We suggest the application of an ACE screening in those professionals who experience anxiety and depressive symptoms related to COVID‐19 care, since they may be more susceptible to presenting a greater psychopathological intensity and functional deterioration.

This study has several limitations. First, it was a cross‐sectional study based on an online questionnaire distributed to health professionals. Generalization of the conclusions is not possible because we did not have a means of approximating the entire population of health professionals. However, the sample size we collected complied with our a priori estimation of the sample size. A longitudinal design is warranted to provide definitive evidence of health workers’ resilience in the face of mental health problems related to COVID‐19. Second, the participants were heterogeneous, and no direct conclusions could be applied to any particular professional group, be it a physicians, nurses, midwives, or others. Third, the participants were from the Lima region; therefore, our findings cannot be generalized to the less‐affected regions of Peru or to professionals from other countries due to cultural and health‐system differences. Fourth, this study was based on a self‐reported questionnaire, and it was not possible to verify mental health problems through structured interviews. Fifth, this study could not explain the difference between pre‐existing mental health symptoms and new symptoms related to COVID‐19 care.

## CONFLICT OF INTEREST

The authors declare no conflict of interest.

### PEER REVIEW

The peer review history for this article is available at https://publons.com/publon/10.1002/brb3.2452

## Data Availability

Data used in this manuscript are available upon request to contact author.
